# Effects of Posteroanterior Thoracic Mobilization on Heart Rate Variability and Pain in Women with Fibromyalgia

**DOI:** 10.1155/2014/898763

**Published:** 2014-05-29

**Authors:** Michel Silva Reis, João Luiz Quagliotti Durigan, Ross Arena, Bruno Rafael Orsini Rossi, Renata Gonçalves Mendes, Audrey Borghi-Silva

**Affiliations:** ^1^Department of Physical Therapy, School of Medicine, Federal University of Rio de Janeiro, 8° Floor 3 (8E-03), Prof Rodolpho Paulo Rocco Street, 21941-913 Rio de Janeiro, RJ, Brazil; ^2^Physical Therapy Division, University of Brasília, QNN 14 Área Especial, Ceilândia Sul, 72220-140 Brasília, DF, Brazil; ^3^Department of Physical Therapy and Integrative Physiology Laboratory, College of Applied Health Sciences, University of Illinois, 1919 W. Taylor Street (MC 898), Chicago, IL 60612, USA; ^4^Healthy-School Unit, Federal University of Sao Carlos, 235 Km. Washington Luis Rodovia, 13565-905 Sao Carlos, SP, Brazil; ^5^Laboratory of Cardiopulmonary Physiotherapy, Federal University of Sao Carlos, 235 Km. Washington Luis Rodovia, 13565-905 Sao Carlos, SP, Brazil

## Abstract

Fibromyalgia (FM) has been associated with cardiac autonomic abnormalities and pain. Heart rate variability (HRV) is reduced in FM with autonomic tone dominated by sympathetic activity. The purpose of this study was to evaluate the effects of one session of a posteroanterior glide technique on both autonomic modulation and pain in woman with FM. This was a controlled trial with immediate followup; twenty premenopausal women were allocated into 2 groups: (i) women diagnosed with FM (*n* = 10) and (ii) healthy women (*n* = 10). Both groups received one session of Maitland mobilization grade III posteroanterior central pressure glide, at 2 Hz for 60 s at each vertebral segment. Autonomic modulation was assessed by HRV and pain by a numeric pain scale before and after the intervention. For HRV analyses, heart rate and RR intervals were recorded for 10 minutes. FM subjects demonstrated reduced HRV compared to controls. Although the mobilization technique did not significantly reduce pain, it was able to improve HRV quantified by an increase in rMSSD and SD1 indices, reflecting an improved autonomic profile through increased vagal activity. In conclusion, women with FM presented with impaired cardiac autonomic modulation. One session of Maitland spine mobilization was able to acutely improve HRV.

## 1. Introduction

Fibromyalgia (FM) is a chronic disorder, which is accompanied by myriad of symptoms such as pain, fatigue, depression, insomnia, and reduced cognitive performance [1]. Chronic fatigue and pain syndromes may precipitate increased sympathetic nervous system activity [2–7]. Specific to the current study, although the exact cause of FM is unknown, some studies suggest autonomic imbalance mechanistically contributes to the symptoms [8, 9].

The autonomic imbalance for FM is characterized by sympathetic hyperactivity at rest and an inability to appropriately respond to physiological stressors [4, 5, 9, 10]. Sympathetic hyperactivity may also be responsible for frequent complaints of cold extremities. Interestingly, a correlation between autonomic dysfunction and symptom severity or quality of life has been previously described [11]. Heart rate variability (HRV) has been used to investigate cardiovascular autonomic modulation as a simple, sensitive, and noninvasive tool [12]. Given the link between abnormal ratios of sympathovagal balance in patients with FM, HRV analysis at rest as well as posttherapeutic interventions may prove to be valuable.

Several therapy strategies [13] have been applied to FM patients with the intention of minimizing the cascade of physically debilitating symptoms. Although there is no consensus, it seems that manual techniques improve quality of life and symptomatology [14]. In a recent study, manual therapy was effective in improving pain intensity, widespread pressure pain sensitivity, impact of FM symptoms on a given patient, sleep quality, and depressive symptoms [15]. The Maitland mobilization is a well-established manual technique that has been applied to a number of musculoskeletal disorders [16].

In relation to HRV, Buttagat et al. [17] demonstrated that manual therapy is effective in increasing cardiac parasympathetic activity, reducing sympathetic activity, and reducing pain and stress in patients with back pain associated with myofascial trigger points. Other authors [18] observed that myofascial trigger-point therapy to the head, neck, and shoulder areas is effective in increasing cardiac parasympathetic activity and improving measures of relaxation.

However, there is no evidence that the Maitland mobilization, focused on mobilizing the thoracic spine, improves autonomic function and pain in FM. The aim of this study was to evaluate the effects of a posteroanterior glide mobilization technique on both HRV and pain. We hypothesize that one session of this manual intervention improves both HRV and pain in woman with FM.

## 2. Methods

### 2.1. Design and Study Population

This was a controlled trial with immediate followup enrolling twenty women. Inclusion criteria were as follows: (1) being in the premenopausal phase and (2) no present history of smoking, lung disease, hypertension, diabetes, hypothyroidism, coronary insufficiency, or other relevant clinical conditions known to affect autonomic control of heart rate, including inflammatory and autoimmune disorders. To the FM group (*n* = 10), the patients were diagnosed with FM by clinicians according to the criteria of the American College of Rheumatology [19]; and the control group (*n* = 10) was composed of apparently healthy women without FM. All subjects were submitted to the following: (1) a clinical assessment (current and past clinical history, family background, lifestyle habits, and physical exam) and (2) physiotherapeutic assessment (postural assessment and muscle tests). The volunteers were informed of experimental procedures and signed an informed consent form before taking part in the study, which was approved by the Ethics Committee of the Federal University of Sao Carlos (109/2006).

### 2.2. Experimental Procedure

Data collection was carried out in an air-conditioned laboratory with a 22°C to 24°C temperature and a 50 to 60 percent relative humidity between 8 a.m. and 12 p.m. The subjects were familiarized with the experimental environment and research personnel. The volunteers were instructed to avoid caffeinated beverages; not to perform physical exercise 24 hours prior to evaluation; have a light meal the morning of data collection; and have an adequate period of sleep the night before (at least 8 hours).

A posteroanterior glide mobilization technique was performed as previously described [20]. The women were instructed to remain in the prone position and an experienced physiotherapist administered the Grade III posteroanterior central pressure glide (III-PAC) at 2 Hz for 60 s at each vertebral segment. Specifically, this manual mobilization was applied between T1 and T12 vertebral structures, corresponding to the thoracic sympathetic preganglionic neurons. The intervention was performed in the control group and FM group by the same physiotherapist that was blinded to each subject's group assignment.

### 2.3. Pain Assessment

Pain was assessed with the numeric pain scale (NPS), which assesses the pain intensity and degree of relief experienced by the patient following an intervention (score of 0 = no pain; 10 = unbearable pain) [21].

### 2.4. Heart Rate and RR Interval Data Acquisition

Heart rate and RR intervals (RRi) were registered beat-to-beat, through a heart rate monitor (Polar S810i) with a 1,000 Hz sampling frequency, fastened by an elastic band to the lower third of the sternum, providing simultaneous transmission to a watch where the data were stored. Afterwards, through a serial port interface and an infrared sensor, data was transported and stored in a personal computer to be analyzed. This assessment was performed at rest in the supine position for 10 min before and after the posteroanterior glide technique. During the data acquisition, the volunteers were instructed to maintain spontaneous breathing to ensure eupneic conditions (the respiratory frequency was monitored). The protocol transition points were also accurately marked to allow for an adequate data analysis.

### 2.5. Signal Processing and HRV Analysis

After acquisition, the signals were transferred to the Polar Precision Performance Software and the section of highest stability for RRi, which included a simple line comprised of at least 256 points, was selected by visual inspection according to the criterion set forth by the Task Force of European Society of Cardiology and the North American Society of Pacing and Electrophysiology [12]. The data were entered into the Kubios HRV Analysis software (MATLAB, version 2 beta, Kuopio, Finland).

Heart rate variability was analyzed by mathematical and statistical models in time and frequency domains and by nonlinear models [19]. In the time domain, the mean RRi, which is all the cyclic components responsible for variability during the recording period and is an estimate of overall HRV, and root mean square of the squares of the differences between successive RRi (rMSSD), in ms, representative of parasympathetic activity, were analyzed. The frequency domain analysis utilized the fast Fourier transform (FFT) on the time series. The application of this algorithm permitted the identification of the power spectral density (PSD) as well as its frequency bands: low frequency (LF) and high frequency (HF), both in normalized units (nu). Two frequency bands that best represent vagal and sympathetic activities of HR control were used in this study. The LF (0.04 to 0.15 Hz) has been attributed to a mixture of sympathetic and parasympathetic modulation, with sympathetic predominance, as well as baroreflex activity. On the other hand, the HF (0.15 to 0.4) has been attributed to parasympathetic activity [12].

For nonlinear analysis, we used Poincaré plot measure indices SD1 and SD2 (the standard derivation of the Poincaré plot perpendicular and along the line of identity, resp.) representative of parasympathetic autonomic activity and total HRV, respectively [22]. Detrended fluctuation analysis (DFA) was also carried out using DFA*α*1 (short-term correlation properties of RRi) and DFA*α*2 (long-term correlation properties of RRi) indices. The technique of the analyses, previously developed by Peng et al. [22], quantifies the presence or absence of fractal-like correlation properties in biological times series and has been used to evaluate the risk of mortality in various groups, given it is a predictor of benign and malignant arrhythmias, sudden cardiac death, and total mortality in patients with reduced left ventricle ejection fraction, acute myocardial infarction, and other cardiovascular diseases [22].

### 2.6. Statistical Analysis

Sample size was determined a priori using G*Power (version 3.1.3; University of Trier, Trier, Germany) with the level of significance set at *P* = 0.05 and power (1 − *β*) = 0.95 in order to detect a large effect (*f* ^2^ > 0.47). We conducted a pilot study with 5 participants to evaluate the effect size for the main dependent variable (rMSSD). Based on these a priori calculations and the pilot study, we set the final sample size at *n* = 20 (10 per group). For continuous data, parametric statistical tests were used given data presented with a normal distribution (Shapiro-Wilk test) and homogenous variances (Levene's test). For demographic and clinical variables, was performed unpaired *t* test. The two-way analysis of variance (ANOVA) assessed the group effect (control versus FM), mobilization effect, and interaction between them. Then, Bonferroni post-hoc was performed to identify differences. All analyses were carried out in SPSS software Release 10.0.1 (Chicago, IL) and all statistical tests with a *P* < 0.05 were considered significant.

## 3. Results

Table 1 lists demographic, anthropometric, clinical characteristics and medication use data for the cohort assessed. We did not observe significant differences between age, weight, height, and body mass index. All women with FM were medicated to manage mood, anxiety, and pain (Table 1).

**Figure 1:**
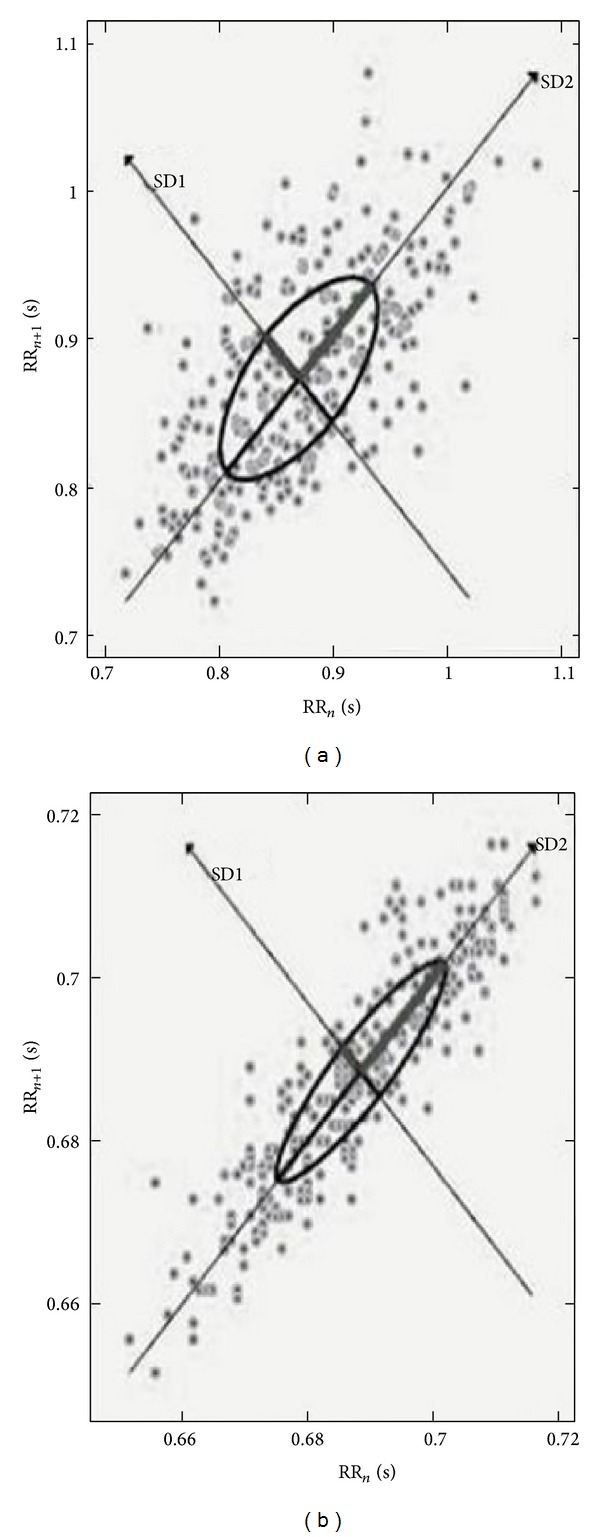
Visual differences in Poincare plot of two women at rest after posteroanterior glide technique mobilization. (a) Healthy woman and (b) woman with fibromyalgia.

Table 2 lists HRV indices of the FM and control groups at rest and after the posteroanterior glide mobilization. The FM group presented with significantly lower rMSSD (*F* = 11.107; *P* = 0.003), HF (nu) (*F* = 2.386; *P* = 0.036), and SD1 (*F* = 10.410; *P* = 0.003) values compared to control at rest. Complementarily, LF (nu) (*F* = 2.836; *P* = 0.016), DFA*α*1 (*F* = 3.151; *P* = 0.088), and DFA*α*2 (*F* = 5.476; *P* = 0.026) were higher in the FM group compared to control. After the manual intervention, rMSSD (*F* = 8.344; *P* = 0.035) and SD1 (*F* = 0.076; *P* = 0.003) indices were significantly higher when compared to baseline only in the FM group. We did not observe significant changes in perceived pain following manual therapy in the FM group.  Figure 1 shows the SD1 and SD2 indices of two women: one of them with FM group and the other in the control group.

## 4. Discussion

To our knowledge, this was the first study to demonstrate the effect of a posteroanterior glide mobilization technique (III-PAC) to the thoracic spine on autonomic modulation in patients with FM.

The main finding of our study was that patients with FM presented important deleterious alterations in HRV at rest suggesting increased sympathetic and decreased parasympathetic activity. Additionally, although manual manipulation did not significantly reduce pain, it was able to significantly improve HRV, as demonstrated by an increase in the rMSSD and SD1 indices (representative of parasympathetic modulation).

Previous studies have shown that FM may be related to changes in autonomic tone, shifting toward an increase in sympathetic activity. Moreover, it has been proposed that dysautonomia is involved in the pathogenesis of FM, which could serve as a mechanism for some of the signs and symptoms associated with this condition [8, 10, 23, 24].

The present study also demonstrates that patients with FM have increased sympathetic activity and decreased activity in the vagal control of HR, demonstrated by linear and nonlinear HRV indices. This sympathetic excitation could contribute to the diffuse pain and tenderness at specific points experienced by patients with FM.

In this context, several interventions have been proposed to minimize the deleterious signs and symptoms caused by FM. Kingsley et al. [25] assessed the acute effects of strengthening exercises on HRV of FM patients and demonstrated lower sympathetic and higher vagal modulation compared to controls after training. Gamber et al. [26] applied chronic osteopathic manipulation in female patients with FM. These patients received craniosacral manipulation for 23 weeks, one treatment for week, for 15 to 30 minutes per session. The authors emphasized that osteopathic manipulation was able to raise pain thresholds, improve comfort levels, effect components related to chronic illness, and increase perceived functional capacity. Castro-Sánchez et al. [27] demonstrated that massage-myofascial release therapy reduces the sensitivity to pain at tender points in patients with FM, improving their pain perception.

Our findings showed that the employed posteroanterior glide technique did not reduce pain. A previous study demonstrated that manual therapy protocol was effective in improving pain intensity [15]. However, the intervention was applied over 5 sessions and the current study assessed the acute effect of a single session on pain, which could help to explain differences in our findings.

However, the potentially significant impact of our findings is the demonstration that only one session of this manual intervention to the thoracic spine was able to modify HRV in women with FM. Considering that there is a correlation between autonomic dysfunction and symptom severity or quality of life [11], these results may represent clinical benefits to patients who suffer from this condition.

In the current investigation, this mobilization technique was performed to each thoracic spine segment, which has an anatomic relationship with the sympathetic chain ganglia. Some studies have shown that spinal manipulation is able to modulate autonomic nervous activity [28, 29]. Yates et al. [19] examined the effect of chiropractic manipulation to T1–T5 spine segments in patients with arterial hypertension. Immediately after the intervention, they observed a reduction in systolic and diastolic blood pressure and anxiety level. A separate case study showed the effects of 10 sessions of chiropractic manipulation (2 sessions per week) applied throughout the spine (C3 to L5) for 6 weeks. After the first session, there was a reduction in sympathetic activity as measured in the band reflecting parasympathetic tone [30].

Interestingly, it was clearly demonstrated in the current investigation that both SD1 and rMSSD significantly increased after one session of manual mobilization in the FM group, which reflects increased parasympathetic activity (Beckers et al., 2006) [31]. The higher vagal activity is an important finding because it may contribute to improvement in vagal-sympathetic balance. Given these findings, it is plausible to hypothesize that the posteroanterior glide technique utilized in the current study may significantly contribute to reducing the debilitating signs and symptoms of FM, improve quality of life, and reduce cardiovascular risk when applied for more than one session.

It is important to recognize a study limitation regarding the acute effect of manual mobilization on pain and HRV and only assessing the outcomes of a single session in FM subjects. Future studies are needed to evaluate the linkage between this and other manual therapy techniques on pain control and autonomic function in patients with FM over multiple treatment sessions. Follow-up studies are also necessary to confirm the long-term effect of spine manual mobilization in FM in order to elucidate the effects after intervention, that is, to observe whether the short-term effects have an accumulative or permanent influence after repeated application in patients with FM. Lastly, patients with FM oftentimes take medications with potential effects on the autonomic nervous system that were not considered in this study. These aspects deserve to be considered in future research.

## 5. Conclusion

In conclusion, the current study observed that women with FM present with altered HRV indices reflecting sympathetic hyperactivity at rest. Additionally, after FM subjects underwent one session of a posteroanterior glide mobilization technique to the thoracic spine, we observed a significant increase in rMSSD and SD1 indices, reflecting an improved autonomic profile through increased vagal activity.

## Figures and Tables

**Table 1:** Demographic, anthropometric, clinical, and medication use data in both groups.

	Fibromyalgia (*n* = 10)	Control (*n* = 10)	*P*
Age, years	52 ± 10	45 ± 9	0.109
Weight, kg	62 ± 9.9	57 ± 6.0	0.321
Height, cm	157 ± 5.0	163 ± 6.0	0.122
Body mass index, kg/m^2^	23.1 ± 3.2	22.6 ± 2.5	0.119
Antidepressant use, %	90	0	<0.001
Anxyolitic use, %	70	0	<0.001
Analgesic use, %	60	0	<0.001
Opiate use, %	10	0	<0.001

Data are presented as mean ± SD or %.

**Table 2:** Pain assessment and linear and nonlinear indices of heart rate variability before and after posteroanterior glide mobilization.

	Fibromyalgia		Control		*P* values		
	Before	After	Before	After	D	M	I
Pain score	6 ± 1	4 ± 1	—	—	—	—	—
HRV indices							
HR, bpm	81 ± 10	77 ± 9	73 ± 9	69 ± 7	ns	ns	ns
iRR, ms	733.5 ± 99.6	749.3 ± 74.6	823.9 ± 99.5	875.6 ± 92.6	ns	<0.05	ns
rMSSD, ms	12.9 ± 6.7	26.3 ± 13.6^†^	34.7 ± 19.0^‡^	37.8 ± 18.7	<0.05	<0.05	ns
SDNN, ms	22.9 ± 13.7	26.3 ± 13.6	40.8 ± 20.0	44.5 ± 15.2	ns	ns	ns
LF, nu	68.6 ± 14.5	51.9 ± 16.9	47.5 ± 8.3^‡^	45.7 ± 13.9	<0.05	ns	ns
HF, nu	31.4 ± 14.5	48.1 ± 16.9	52.4 ± 8.3^‡^	54.2 ± 13.9	<0.05	ns	ns
LF/HF	3.2 ± 3.0	1.4 ± 1.4	0.5 ± 0.3	0.9 ± 0.6	ns	ns	ns
SD1, ms	9.19 ± 4.7	11.0 ± 5.3^†^	24.2 ± 14.0^‡^	26.7 ± 13.2	<0.05	<0.05	ns
SD2, ms	31.0 ± 18.9	35.3 ± 19.1	52.3 ± 26.3	56.9 ± 17.9	ns	ns	ns
DFA*α*1	1.2 ± 0.1	1.1 ± 0.1	1.0 ± 0.1^‡^	0.95 ± 0.2	<0.05	ns	<0.05
DFA*α*2	1.0 ± 0.1	0.9 ± 0.1	0.71 ± 0.2^‡^	0.93 ± 0.2	<0.05	ns	<0.05

Data are presented as mean ± standard deviation. Two-way ANOVA test with Bonferroni post-hoc test. D: disease effect; M: mobilization effect; I: interaction; ns: nonsignificant. ^†^ *P* < 0.05: before versus after posteroanterior glide technique; ^‡^ *P* < 0.05: fibromyalgia versus control.

## References

[1] Wolfe F, Smythe HA, Yunus MB (1990). The American College of Rheumatology 1990. Criteria for the classification of fibromyalgia. Report of the Multicenter Criteria Committee. *Arthritis and Rheumatism*.

[2] Cohen H, Neumann L, Shore M, Amir M, Cassuto Y, Buskila D (2000). Autonomic dysfunction in patients with fibromyalgia: application of power spectral analysis of heart rate variability. *Seminars in Arthritis and Rheumatism*.

[3] Cohen H, Neumann L, Alhosshle A, Kotler M, Abu-Shakra M, Buskila D (2001). Abnormal sympathovagal balance in men with fibromyalgia. *Journal of Rheumatology*.

[4] Martínez-Lavín M, Hermosillo AG, Mendoza C (1997). Orthostatic sympathetic derangement in subjects with fibromyalgia. *Journal of Rheumatology*.

[5] Martínez-Lavín M, Hermosillo AG (2000). Autonomic nervous system dysfunction may explain the multisystem features of fibromyalgia. *Seminars in Arthritis and Rheumatism*.

[6] Meeus M, Goubert D, de Backer F (2013). Heart rate variability in patients with fibromyalgia and patients with chronic fatigue syndrome: a systematic review. *Seminars in Arthritis and Rheumatism*.

[7] Raj SR, Brouillard D, Simpson CS, Hopman WM, Abdollah H (2000). Dysautonomia among patients with fibromyalgia: a noninvasive assessment. *Journal of Rheumatology*.

[8] Fishman M, Jacono FJ, Park S (2012). A method for analyzing temporal patterns of variability of a time series from Poincaré plots. *Journal of Applied Physiology*.

[9] Reyes del Paso GA, Garrido S, Pulgar Á, Duschek S (2011). Autonomic cardiovascular control and responses to experimental pain stimulation in fibromyalgia syndrome. *Journal of Psychosomatic Research*.

[10] Staud R (2008). Heart rate variability as a biomarker of fibromyalgia syndrome. *Future Rheumatology*.

[11] Solano C, Martinez A, Becerril L (2009). Autonomic dysfunction in fibromyalgia assessed by the composite autonomic symptoms scale (COMPASS). *Journal of Clinical Rheumatology*.

[12] Malik M (1996). Heart rate variability: standards of measurement, physiological interpretation, and clinical use. *Circulation*.

[13] Bronfort G, Haas M, Evans R, Leininger B, Triano J (2010). Effectiveness of manual therapies: the UK evidence report. *Chiropractic and Osteopathy*.

[14] Terhorst L, Schneider MJ, Kim KH, Goozdich LM, Stilley CS (2011). Complementary and alternative medicine in the treatment of pain in fibromyalgia: a systematic review of randomized controlled trials. *Journal of Manipulative and Physiological Therapeutics*.

[15] Castro-Sánchez AM, Aguilar-Ferrándiz ME, Matarán-Peñarrocha GA, Sánchez-Joya MD, Arroyo-Morales M, Fernández-de-Las-Peñas C (2014). Short-term effects of a manual therapy protocol on pain, physical function, quality of sleep, depressive symptoms and pressure sensitivity in women and men with fibromyalgia syndrome: a randomized controlled trial. *The Clinical Journal of Pain*.

[16] Hurley DA, McDonough SM, Baxter GD, Dempster M, Moore AP (2005). A descriptive study of the usage of spinal manipulative therapy techniques within a randomized clinical trial in acute low back pain. *Manual Therapy*.

[17] Buttagat V, Eungpinichpong W, Chatchawan U, Kharmwan S (2011). The immediate effects of traditional Thai massage on heart rate variability and stress-related parameters in patients with back pain associated with myofascial trigger points. *Journal of Bodywork and Movement Therapies*.

[18] Delaney JPA, Leong KS, Watkins A, Brodie D (2002). The short-term effects of myofascial trigger point massage therapy on cardiac autonomic tone in healthy subjects. *Journal of Advanced Nursing*.

[19] Yates RG, Lamping DL, Abram NL, Wright C (1988). Effects of chiropractic treatment on blood pressure and anxiety: a randomized, controlled trial. *Journal of Manipulative and Physiological Therapeutics*.

[20] Maitland GD (2000). *Vertebral Manipulation*.

[21] Borg G (1998). *Borg’s Perceived Exertion and Pain Scales*.

[22] Peng C-K, Havlin S, Hausdorff JM, Mietus JE, Stanley HE, Goldberger AL (1995). Fractal mechanisms and heart rate dynamics: long-range correlations and their breakdown with disease. *Journal of Electrocardiology*.

[23] Castro-Sánchez AM, Matarán-Peñarrocha GA, Sánchez-Labraca N, Quesada-Rubio JM, Granero-Molina J, Moreno-Lorenzo C (2011). A randomized controlled trial investigating the effects of craniosacral therapy on pain and heart rate variability in fibromyalgia patients. *Clinical Rehabilitation*.

[24] Mostoufi SM, Afari N, Ahumada SM, Reis V, Wetherell JL (2012). Health and distress predictors of heart rate variability in fibromyalgia and other forms of chronic pain. *Journal of Psychosomatic Research*.

[25] Kingsley JD, Panton LB, McMillan V, Figueroa A (2009). Cardiovascular autonomic modulation after acute resistance exercise in women with fibromyalgia. *Archives of Physical Medicine and Rehabilitation*.

[26] Gamber RG, Shores JH, Russo DP, Jimenez C, Rubin BR (2002). Osteopathic manipulative treatment in conjunction with medication relieves pain associated with fibromyalgia syndrome: results of a randomized clinical pilot project. *Journal of the American Osteopathic Association*.

[27] Castro-Sánchez AM, Matarán-Pearrocha GA, Granero-Molina J, Aguilera-Manrique G, Quesada-Rubio JM, Moreno-Lorenzo C (2011). Benefits of massage-myofascial release therapy on pain, anxiety, quality of sleep, depression, and quality of life in patients with fibromyalgia. *Evidence-Based Complementary and Alternative Medicine*.

[28] Sterling M, Jull G, Wright A (2001). Cervical mobilisation: concurrent effects on pain, sympathetic nervous system activity and motor activity. *Manual Therapy*.

[29] Wright A (1995). Hypoalgesia post-manipulative therapy: a review of a potential neurophysiological mechanism. *Manual Therapy*.

[30] Driscoll MD, Hall MJ (2000). Effects of spinal manipulative therapy on autonomic activity and the cardiovascular system: a case study using the electrocardiogram and arterial tonometry. *Journal of Manipulative and Physiological Therapeutics*.

[31] Beckers F, Verheyden B, Aubert AE (2006). Aging and nonlinear heart rate control in a healthy population. *The American Journal of Physiology—Heart and Circulatory Physiology*.

